# The Validity and Reliability of the Short Form of the Adolescent Stress Questionnaire (ASQ-14) Among Greek Adolescents Aged 15–18

**DOI:** 10.3390/children12111537

**Published:** 2025-11-14

**Authors:** Ntina Kourmousi, Kalliopi Kounenou, Christos Pezirkianidis, Antonios Kalamatianos, George P. Chrousos

**Affiliations:** 1 Department of Education, School of Pedagogical and Technological Education, 15122 Marousi, Greece; kkounen@aspete.gr; 2 Laboratory of Psychological Interventions in Education, School of Pedagogical & Technological Education, ASPETE, 15122 Marousi, Greece; 3 Department of Psychology, Panteion University of Social and Political Sciences, 17671 Athens, Greece; pezir@panteion.gr; 4 Department of Education, University of Nicosia, Nicosia 2417, Cyprus; kalamatianos.a@unic.ac.cy; 5 Center for Adolescent Medicine and UNESCO Chair in Adolescent Health Care, First Department of Pediatrics, Medical School, National and Kapodistrian University of Athens, Aghia Sophia Children’s Hospital, 11527 Athens, Greece; chrousge@med.uoa.gr; 6 University Research Institute of Maternal and Child Health and Precision Medicine, National and Kapodistrian University of Athens, Aghia Sophia Children’s Hospital, 11527 Athens, Greece

**Keywords:** stress, adolescence, Adolescent Stress Questionnaire, validity, students, Greece

## Abstract

**Highlights:**

**What are the main findings?**

- The Greek ASQ-14 demonstrated acceptable reliability. Greek adolescents with high perceived socio-economic status had significantly greater scores in external stress factors and significantly lower scores in internal ones.
- Female adolescents had significantly greater scores in ASQ.

**What is the implication of the main finding?**

- The Greek ASQ-14 responds to the necessity of more viable, concise, and user-friendly stress-assessing instruments for Greek adolescents.

**Abstract:**

Background: Stress has devastating consequences for adolescents’ physical and mental health. Therefore, having the tools to accurately measure it is of critical importance. This paper seeks to evaluate the psychometric characteristics of the Greek version of the 14-item Adolescent Stress Questionnaire (ASQ-14), which measures adolescents’ daily experience of common stressors. Methods: The study questionnaire was administered to 176 male and female 15- to 18-year-old students, coming from several regions of Greece. The psychometric characteristics of the Greek ASQ-14 were investigated by confirmatory factor analysis(CFA) and exploratory factor analysis (EFA). The Kaiser–Meyer–Olkin (KMO) procedure was applied for measuring sample adequacy. Principal axis factoring (PAF) was chosen as the extraction method and Varimax rotation was used. Internal consistency was evaluated using Cronbach’s alpha coefficient. Scale intercorrelations were evaluated via Pearson’s r correlation coefficient. The discriminant construct validity was evaluated by analyzing the differences in the ASQ-14 scales between girls and boys, using Student’s *t*-tests. Results: Two factors emerged: External Pressures and Constraints and Internal Stress and Uncertainty explained 18.5% and 14.5% of the variance, respectively. The Greek ASQ-14 demonstrated acceptable reliability. Concerning discriminant validity, participants with high perceived socioeconomic status had significantly greater scores in “External Pressures & Constraints” and significantly lower scores in “Internal Stress & Uncertainty, while girls had significantly greater scores in both scales and greater total scores than boys. Conclusions: The Greek ASQ-14 emerges as a psychometrically sound tool for screening stress among Greek adolescents.

## 1. Introduction

### 1.1. Stress in Adolescence

Adolescents are subject to stress when they interpret a situation as demanding, distressing, or threatening and believe they lack the resources to cope effectively. Stress can be defined as “…the condition that results when person or environment transactions lead the individual to perceive a discrepancy between the demands of a situation and the resources of the person’s biological, psychological and social systems” [1] (p. 128). It arises when homeostasis is, or is perceived to be, under threat [2].

Adolescence is widely recognized as a particularly stressful life stage and is characterized by remarkable plasticity and significant changes in virtually every aspect of an individual’s life; psychological and physiological transformations include brain and body maturation, heightened sensitivity to social and peer relationships, a growing need for independence, and emotional intensity [3]. Major relationship stressors such as the perception of poor support from family, peers, and teachers [4], and other stress factors such as body image anxiety [5], concerns about the future [6], academic stress [7], and socioeconomic adversity [8] contribute to elevated stress levels among adolescents [9] and frequently lead to psychological distress and ineffective coping mechanisms [8]. However, it is not the stressors in themselves that are harmful, but rather the lack of resources that would allow one to cope with them successfully [10].

Stressful life events have been found to predict children’s and adolescents’ mental health problems [11]. Stress has a substantial impact on adolescents’ physical and psychosocial health, shaping stress reactivity [12] and contributing to various psychological dysfunctions like depression, anxiety, and substance abuse, which are frequently observed during this phase of development [13]. Furthermore, exposure to stressful life situations during adolescence has been associated with increased perceived stress, which affects both short- and long-term wellbeing [14].

### 1.2. Assessment of Stress in Adolescence

Given these concerns, accurately measuring adolescent stress is essential for the planning of primary health care and educational services, and demands valid and reliable instruments [10]. Generally, there are three approaches in stress assessment. Firstly, there is the biological or physiological one that uses neuroendocrine biomarkers—such as glucocorticoids and catecholamines—and immunological markers like cytokines, CRP, and IGF-1 [15].

Secondly there is the environmental approach that uses tools which assess stressful life events [15]. Concerning adolescent populations, such measures include:

(a) The Adolescent Perceived Events Scale (APES) [14]: This tool examines positive and negative stressful events experienced during the past six months and has been validated exclusively for use among English- and Spanish-speaking adolescents [16].
(b) The Adolescent Stress Questionnaire (ASQ) [17]: Originally developed to assess stress-inducing situations in Australian adolescents, the ASQ has been translated and validated in multiple languages, including Spanish, Dutch, Norwegian, Turkish, and Hungarian [16]. Its long version was also validated in a study in 1240 adolescents from Germany, Hungary, Spain, Greece, and Denmark [18]. Greek versions of the long form, with 58 items [19], and of the 27-item short form [20] of the tool are available.
(c) The Perceived Stressors Global Scale for Adolescents (PSGS-A) [21]: This more recent tool evaluates six categories of stressful events—social pressure, academic stressors, family concerns, social exhibition, daily hassles, and critical incidents. Currently, it has only been validated in a Mexican adolescent population [21].

Thirdly there is the psychological stress assessment approach that uses self-report psychometric tools which measure perceived stress [15]. Regarding adolescents, such instruments include:

(a) The Perceived Stress Scale (PSS) [22]: This instrument measures how individuals, both adults and adolescents, perceive stress over the preceding four weeks. According to Cohen’s Laboratory for the Study of Stress, Immunity, and Disease, the PSS is suitable for population samples of at least a junior high school education [16]. It has been translated into 25 languages, including Greek, and validated for use in Greek adolescents [23,24,25].
(b) The Stress Appraisal Measure for Adolescents (SAMA), a 14-item questionnaire that measures primary and secondary cognitive appraisal of stress in youth of 14–18 years old, by the use of a 5-point Likert scale [26].
(c) The Impact of Event Scale for adolescents (IES) [27], which is a self-report instrument that assesses current subjective distress and identifies post-traumatic stress disorder (PTSD) symptoms.

### 1.3. The Purpose of the Present Study

To date, the Adolescent Stress Questionnaire, both the long-form and 27-item ones, remain the only validated environmental approach stress assessment tools that allow Greek adolescents disclose the stressors they face and the level of strain resulting from them [19]. This gap highlights the need for more reliable, brief, and accessible instruments to assess stress in Greek adolescents. Both the ASQ original form and its shortened 27-item form have been used in Greek populations, mostly concerning validation studies, e.g., [18,19,20]. However, despite the fact that the ASQ has been translated into many languages (e.g., Norwegian, Dutch, Hungarian, Spanish, Swedish, Turkish, and Greek) and used worldwide, results regarding its psychometric properties are mixed [20]; empirical studies conducted in Australia [17], Greece [19], New Zealand [28], and Spain [29], have provided support for the adequacy of the factor structure in the long version of the instrument. Conversely, other investigations have raised substantial concerns regarding its structural validity and internal consistency [30], as well as factor loadings [31]. Nevertheless, the predominant body of research characterizes the ASQ as a psychometrically robust and culturally adaptable instrument for assessing stress [20]. Moreover, the availability of abbreviated forms further enhances its utility, while the length of the original long ASQ version remains a practical concern for researchers, especially when it comes to its use in projects that include multiple instruments [32] and in screening stressors in large samples [33]. Concerning the shortened version with 27 items, it does not cover all ten scales of the original ASQ, since the “Stress of emerging adult responsibility” subscale’s items are omitted [33]. Therefore, there would be plenty of benefits to using the ASQ short form of 14 items for the assessment of adolescents’ stressors [33]: it facilitates screening and analyses of stress with other variables, enables the quick carrying out of assessments, increases the likelihood of completion, and provides comprehensive data. The present study aims to evaluate the psychometric properties of the Greek version of the ASQ-14, for use among adolescents aged 15 to 18 years. We hypothesized that the Greek version of the ASQ-14 would have sound psychometric properties. The validation study will offer researchers a Greek version of a short environmental-approach adolescent stress-assessment instrument, well-suited for both research and clinical settings, owing to its brevity and efficiency of administration. Furthermore, the study findings will enhance Greek research concerning the identification, understanding, and prevention of stressors and stress load among adolescents.

## 2. Materials and Methods

### 2.1. Study Design, Setting, and Participants

The empirical study of the instrument’s psychometric properties using descriptive and exploratory methods took place between April and May 2023. It was conducted by the School of Pedagogical and Technological Education (ASPETE—A.Σ.ΠAΙ.Τ.Ε.)—which is a Greek university that trains secondary school educators—in collaboration with the University of West Attica and the UNESCO Chair on Adolescent Health Care. The study received approval from the Ethics and Conduct Committee of ASPETE (Protocol no: 23-4/4/23).

Eligibility for inclusion required participants to be 15- to 18-year-old students of any of the three grade levels of Greek Lyceums (upper secondary schools). Additionally, students needed to have proficiency in reading, writing, and understanding Greek.

Participants were recruited with the use of an online invitation to the parents of Lyceum students, issued by the study organizers, namely ASPETE, the University of West Attica, and the UNESCO Chair on Adolescent Health Care. The invitation was sent to all Parents’ Associations, and also to parents throughout Greece, via the Hellenic Federation of the Teachers of Tutoring Centers (OΕΦΕ). This invitation presented the purpose of the study, detailed the measures taken to ensure participant anonymity, and included a link to the study questionnaire for parents who consented to their child’s voluntary participation. Students were informed about the study organizers, scope, and time completion (approx. 15 min) on the opening page of the questionnaire. They were able to complete it at home, at their own time of convenience. Upon completion they could download stress-management instructions and techniques for teenagers. A total of 176 high school students from different regions of Greece was considered enough to form the study sample, according to the commonly used “ten participants per item” rule.

### 2.2. Measures

The study questionnaire required demographic data, such as sex, age, region, etc.; socioeconomic data, such as parents’ profession, parental level of studies, perceived family socioeconomic status, etc.; and school data, such as attending general or special education, general or vocational Lyceum, grade, etc., as well as data regarding students’ daily habits such as routines, type of diet, having breakfast, etc. It also included the Greek version of the long form of the Adolescent Stress Questionnaire (58 items), which was already validated and found to have sound psychometric abilities for use in Greek adolescents [19].

The long version of the Adolescent Stress Questionnaire (ASQ) comprises 58 items that investigate adolescents’ daily experience of common stressors, categorized in situations across 10 key domains: (i) family environment, (ii) school performance, (iii) school attendance, (iv) romantic relationships, (v) pressure by peers, (vi) interaction with teachers, (vii) uncertainty for the future, (viii) conflicts in the school or leisure environment, (ix) financial strain, and (x) emerging adult responsibility. The ASQ allows adolescents to report the extent to which any such recent stressor has been a psychological pressure for them, by responses recorded on a 5-point Likert scale, ranging from 1 (Not at all stressful) to 5 (Very stressful), with higher scores indicating greater levels of stress [17]. In the present study, a short form of the scale—namely the ASQ-14 [33]—was selected for validation, in order to assess its psychometric properties. The ASQ-14 was created by Blanca and colleagues and consists of 14 items deriving from all ten scales of the original ASQ [33]. No translation was necessary since the ASQ-14 items were all included in the Greek version of the ASQ-58 [19].

### 2.3. Statistical Analysis

Quantitative variables were presented as mean values (SD), whereas qualitative variables were summarized as absolute and relative frequencies.

A confirmatory factor analysis (CFA) with maximum likelihood procedure was conducted in order to test the unidimensional structure of ASQ-14. The fit of the CFA model was assessed by the use of the comparative fit index (CFI), the Tucker–Lewis Index (TLI), SRMR (Standardized Root Mean Squared Error), and the root mean square error of approximation (RMSEA) [34]. The guidelines for interpreting the fit of a model based on these indicators are several [35,36,37]. Regarding the CFI and TLI indices, values that are close to or greater than 0.90 are considered to express a good fit to the data. RMSEA values of less than 0.05 reflect a good fit, and values as high as 0.08 indicate a reasonable one. SRMR values of less than 0.08 show a good fit.

Since the questionnaire’s original structure was not found satisfactory, an exploratory factor analysis (EFA) was conducted in order to evaluate the tool’s construct validity, disclose possible underlying structures, and reduce the number of variables. A Kaiser–Meyer–Olkin (KMO) procedure for measuring sample adequacy was also applied. The cut-off point for factor loadings was 0.40, and for Eigenvalues 1.00. Principal axis factoring (PAF) was chosen as the extraction method. Oblimin rotation was applied initially, but since the correlation between the latent variables was found to be low (i.e., under 0.30), Varimax rotation was used. The internal consistency reliability was examined by means of Cronbach’s alpha analysis. Scales of reliability equal to or greater than 0.70 were considered acceptable. The structure that emerged from the exploratory factor analysis was checked further via confirmatory factor analysis.

Scale intercorrelations were evaluated via Pearson’s r correlation coefficient. The discriminant construct validity was evaluated by analyzing the differences in ASQ-14 scales between girls and boys, and between low and high perceived family SES, using Student’s *t*-tests.

All the reported *p* values were two-tailed, with statistical significance set at *p* < 0.05. Data analyses were performed by the use of SPSS statistical software (version 27.0) and STATA (version 15).

## 3. Results

### 3.1. Demographic Characteristics of the Sample

The sample consisted of 176 Greek students, with a mean age of 17.1 years (SD = 0.8 years). Their characteristics are shown in Table 1.

The majority of the sample were girls (74.4%), and attended the third grade (57.4%) of general Lyceum (upper high school) education (94.8%). Also, 90.6% had siblings, and 86.2% were living with both of their parents. Most of the students characterized their family socioeconomic status as middle (75.6%) and systematically followed a daily program (routine) in everyday life (88.6%).

### 3.2. ASQ-14 Items

The 14 items of the ASQ-14 are described analytically in Table 2.

For half of our sample students, concerns about their future appeared to be a major stress factor during the past year. Also, 37.5% of the students found the lack of understanding by their parents very stressful. Furthermore, 48.9% of the students did not find not having enough time for their boy/girlfriend stressful, while 44.9% did not find it stressful to have disagreements between them and their teachers.

### 3.3. Factor Analyses

**Figure 1:**
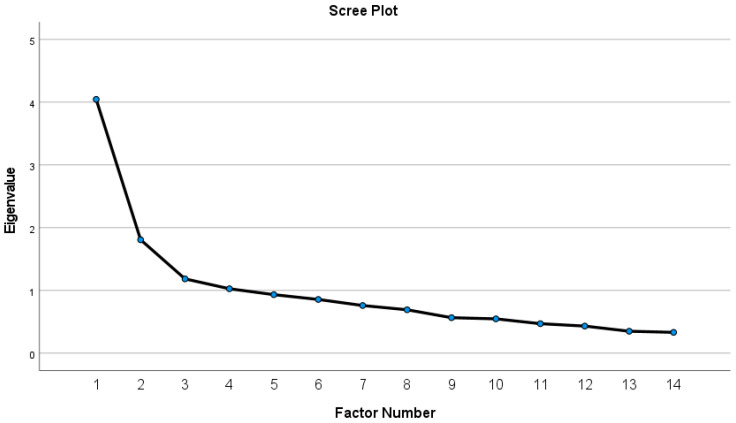
Scree plot.

A confirmatory factor analysis was conducted in order to check the unidimensional structure proposed by the literature. This structure was found unsatisfactory, since all indices were out of the acceptable ranges (CFI = 0.67; TLI = 0.61; RMSEA = 0.120; SRMR = 0.100). Thus, exploratory factor analysis was applied, with Varimax rotation, after evaluating the data and finding it appropriate to perform such an analysis (KMO = 0.78; χ^2^ = 610.92; *p* < 0.001 for Bartlett’s test). The scree plot suggested three scales (Figure 1). However, the third factor consisted of only two items, so this factor was discarded, following the guidelines of Raubenheimer [38], and the analysis was rerun by limiting the factors to two. The results are presented in Table 3.

**Figure 2:**
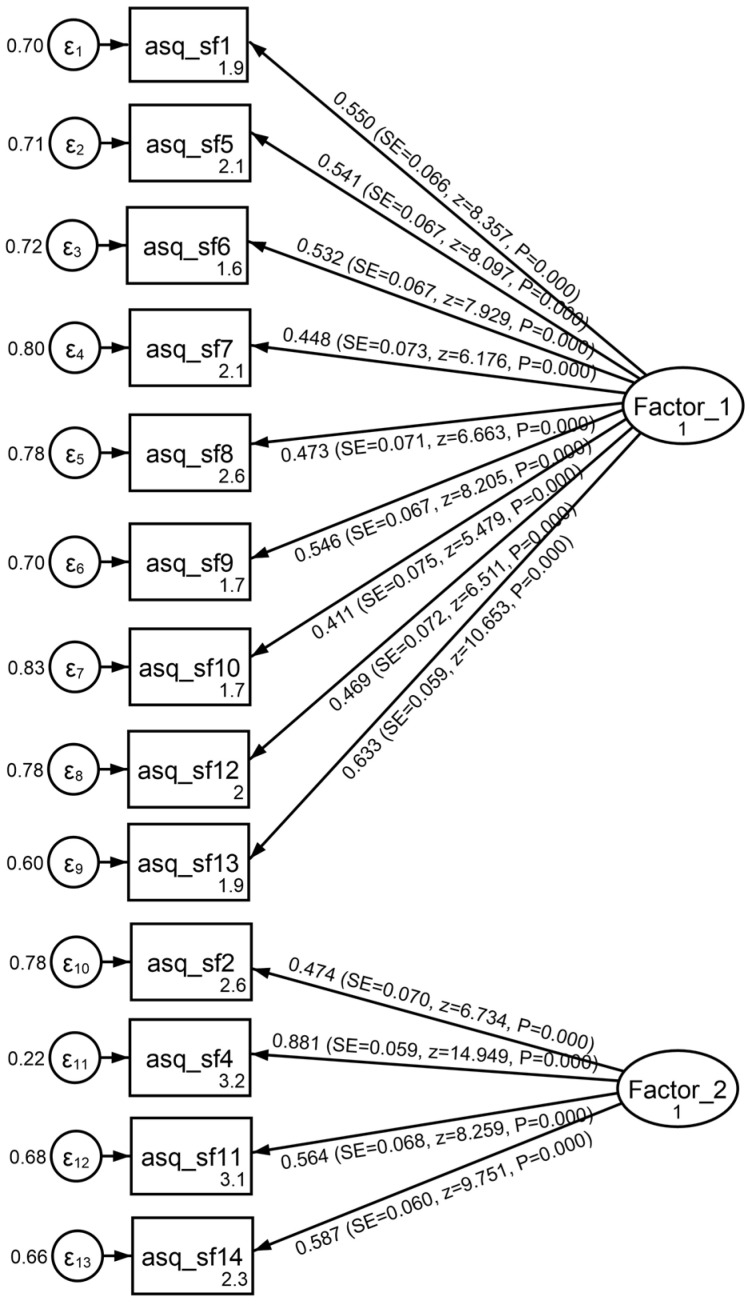
Path diagram for ASQ-14.

Two factors emerged explaining 33.0% of the total variance. More analytically, the first factor (labeled: External Pressures and Constraints) included nine items (1, 5, 6, 7, 8, 9, 10, 12, 13) and explained 18.5% of the variance. The second factor (labeled: Internal Stress and Uncertainty) included four items (2, 4, 11, 14) and explained 14.5% of the variance. Item 3 had a loading lower than 0.40 in both factors; thus, it was not grouped in any of the factors. For the rest of the items, their communalities were at least 0.40, and factor loadings ranged from 0.40 to 0.84, supporting the stability of the extracted factor structure. The structure found in the exploratory analysis was also checked via confirmatory factor analysis, and it was found to be within an acceptable range (CFI = 0.90; TLI = 0.84; RMSEA = 0.078; SRMR = 0.079). The path diagram is provided in Figure 2.

### 3.4. Internal Consistency and Intercorrelations

Cronbach’s alpha coefficients and item-total correlation coefficients for each factor are presented in Table 4.

All item-total correlation coefficients were over 0.30, and all reliability indices were at least 0.70. The removal of any of the items within each factor did not significantly change the factor’s reliability index; thus, no item needed to be removed. The overall reliability index was 0.80, indicating acceptable reliability.

Descriptive measures and intercorrelations of the ASQ-14 scales are provided in Table 5.

The mean score in External Pressures and Constraints was 24.44 (SD = 7.38), in Internal Stress and Uncertainty it was 14.19 (SD = 3.74), and the mean total score was 42.13 (SD = 10.09). Significant and positive correlations were found among the two ASQ-SF subscales (r = 0.38; *p* < 0.001), as well as between all subscales and the total score (r = 0.67, *p* < 0.001 for Internal Stress and Uncertainty; r = 0.92, *p* < 0.001 for External Pressures and Constraints).

### 3.5. Discriminant Validity

In terms of discriminant validity, the ASQ-14 scales were compared between participants with high and low perceived family socioeconomic status (Table 6) and between girls and boys (Table 7).

**Figure 3:**
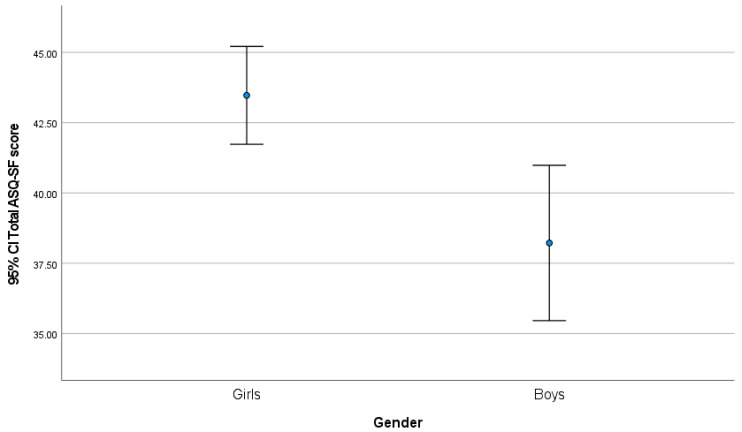
Total ASQ-14 score by sex.

Girls had significantly greater scores on both scales, compared to boys. Also, girls had significantly greater total scores than boys (*p* = 0.002; Figure 3).

## 4. Discussion

The presented study is the first validation study of the Greek version of ASQ-14 in a middle adolescent population. The findings indicate that the Greek ASQ-14 provides a brief and dependable measure for future research on Greek adolescents. The original one-factor structure of the scale was not found satisfactory by CFA, so exploratory factor analysis (EFA) with Varimax rotation was used. Our study results show that the structure of the Greek ASQ-14 scale comprises two factors, labeled (a) External Pressures and Constraints and (b) Internal Stress and Uncertainty. The categorization of stressors into external and internal has been used, in general, in the literature concerning adolescent—e.g., [39]— and adult—e.g., [40,41]—stress. The 14-item ASQ has not been used in other validation studies; thus, its one-factor structure has not been confirmed by other researchers. However, there are cases where the factor structure of other ASQ versions has been moderate—e.g., [18]—or not confirmed—e.g., [20,32]. The study’s highest factor loadings in our study appeared in the items a) having difficulty with some subjects and b) having concerns about the future. This finding contradicts that of the authors of the original ASQ-14, who found the highest factor loadings corresponded to the items “lack of understanding by your parents” and “arguments at home” [33]. It also contradicts the findings of Darviri and colleagues’ [19] study concerning the ASQ-58 Greek validation, where the item “lack of freedom” appeared to have the highest factor loading. Nevertheless, having difficulty with subjects can indeed cause a lot of stress to most Greek middle adolescents, since university entrance seems to be not only their own main concern, but their family’s as well [42]; in Greece, the lead-up to the Panhellenic exams is characterized by intense academic pressure and emotional fatigue [42,43]. As for the high factor loading of the item “having concerns about the future”, studies also emphasize that uncertainty about future careers, education, and societal pressures adds to stress, with many adolescents feeling anxious about whether they will be able to handle upcoming responsibilities [6,44]. Another reason for the shift in teen stressors during times of abundance from “lack of understanding”, “arguments at home”, and “lack of freedom” to “having difficulty in school subjects” and “having concerns about the future” during post-Greek financial crisis and also post-COVID-19 crisis times, is that it might also reflect a shift in pressures, according to our opinion. As MacDonald and colleagues [45] state concerning the post-COVID-19 youth, “amidst very rapidly changing political and economic circumstances, continuing precarity for young people seems to be one certainty” [45] (p. 723).

Regarding score reliability, the total scale demonstrated an internal consistency coefficient of 0.80, reflecting adequate reliability, a finding in line with that of the authors of the original ASQ-14 [33], that of De Vriendt and colleagues concerning the original version [18], and that of Ertanir and colleagues regarding the 27-item version [20]. However, this value is lower than the coefficient of 0.95 found by Lima et al. [29] in the ASQ long version, probably due to the smaller number of items, as Blanca and her colleagues explain [33]. All item-total correlation coefficients were over 0.30, which are therefore adequate, as also found by the authors of the original ASQ-14 [25].

For the scale’s discriminant validity, we used perceived family SES, as well as gender, since both those factors’ associations with adolescents’ perceived stress have been well established (e.g., all). Our study results showed that adolescents who felt that their families were of high socioeconomic status had significantly greater scores in “External Pressures and Constraints” and significantly lower scores in “Internal Stress and Uncertainty”. Other researchers have found that subjective socioeconomic status significantly impacts adolescent stress [46] and mental health [47]. Analytically, it has been found that socioeconomic status impacts the brain [48,49], and more specifically, the volume of the hippocampus [50] and amygdala [51]. Concerning Greece, families—especially those of high status—put a high amount of pressure on adolescents during their last years of secondary education, urging them to work as hard as they can to enter university [42]. This fact can explain our finding concerning the high scores of Greek adolescents who feel that their families are of high socioeconomic status in “External Pressures and Constraints”. On the other hand, the same children scored lower in “Internal Stress and Uncertainty”, probably because the perceived high socioeconomic status makes them feel safer regarding concerns about their future, as has been shown not only for Greek adolescents [52], but for adolescents coming from other countries too [53,54]. The study also showed significant differences between boys and girls, with girls scoring higher in both domains and having greater total scores compared to boys. This finding could be impacted by the fact that the majority of the study sample consisted of females; however, it is consistent with the findings of the original ASQ-14 [25], as well as with those of previous researchers [17,18,28,55]. More specifically, research has consistently highlighted notable gender differences in the sources and impacts of stress among adolescents. Female adolescents frequently report higher levels of stress in contexts involving family and peer interactions, where girls tend to perceive negative interpersonal events as more stressful [56]. Furthermore, studies have found that girls often experience greater academic stress than boys [57]. Factors such as increased school workload, emotional factors, and societal expectations have been identified as underlying contributors to this difference [58].

## 5. Study Limitations and Recommendations for Future Studies

This study has several limitations. Given the relatively limited sample size and the predominance of female participants, the findings cannot be generalized to the entire adolescent population. However, as the study invitation was sent to parents of adolescents across Greece, the sample included students from all regions of the country. The majority were from Attica and Central Macedonia, home to the capital and co-capital cities. According to the Hellenic Statistical Authority, Attica alone accounts for 37.7% of Lyceum students in Greece (www.statistics.gr (accessed on 10 June 2025), https://www.statistics.gr/documents/20181/8fa5c436-3169-54c0-3ad1-1474a9caef0e (accessed on 10 June 2025)), a figure close to the one reflected in our sample. Additionally, the study encompassed students from a wide range of socioeconomic backgrounds. Concerning the study’s small sample size, although it meets the conventional “ten participants per item” guideline, we acknowledge that this rule is only a general heuristic. The adequacy of the sample size for factor analysis also depends on characteristics such as item communalities, factor loadings, and the number of factors extracted. While our data showed acceptable communalities and factor loadings, future studies with larger and more diverse samples are warranted to confirm the stability and generalizability of the factor structure. Another limitation is that test–retest reliability was not evaluated, and the study relied solely on self-reported measures with a negative valence. Additionally, the study did not evaluate the stability of the ASQ-14 over time, and the use of a cross-sectional design does not make it possible to infer the direction of causality between the variables. Despite these limitations, the Greek version of the ASQ-14 demonstrated satisfactory validity and reliability, supporting its use in both research and health care settings. Future research should extend the validation of the ASQ-14 to younger adolescents through prospective study designs. Furthermore, incorporating qualitative data on adolescents’ experiences and narratives of stress, as well as examining the relationship between stress and neuroendocrine biomarkers, would provide valuable insights.

## 6. Conclusions

The results of this study suggest that the ASQ-14 serves as a useful rapid psychometric instrument for health professionals in evaluating stress and identifying minor causes of stress during adolescence. As stated by the instrument’s authors, the ASQ-14 could be employed in large adolescent samples to identify those who suffer high levels of stress and are therefore considered at risk and to be targets for detailed assessment [25]. Additionally, the findings underscore the intricate relationship between stressful life situations and adolescent stress levels, reinforcing the importance of effective coping mechanisms and robust social support systems. Facilitating access to counseling services and introducing school-based, ecological interventions may help reduce the adverse effects of stressors on adolescent mental health [59].

## Figures and Tables

**Table 1:** Sample’s characteristics.

*n* = 176		*n* (%)
Gender		
	Girls	131 (74.4)
	Boys	45 (25.6)
Grade		
	1st upper high school (Lyceum)	17 (9.7)
	2nd upper high school (Lyceum)	58 (33.0)
	3rd upper high school (Lyceum)	101 (57.4)
Type of upper high school (Lyceum)		
	General	164 (94.8)
	Vocational	7 (4.0)
	Experimental	1 (0.6)
	Pilot	1 (0.6)
Learning difficulty		16 (9.1)
Siblings		154 (90.6)
Living with both parents		150 (86.2)
Perceived family socioeconomic status		
	Low	11 (6.3)
	Middle	133 (75.6)
	High	32 (18.2)
Follow systematically a program in everyday life		156 (88.6)
Involvement with a sport or type of sport for at least 2 h a week		74 (42.0)
Other activities or hobbies		90 (51.1)
Region		
	Eastern Macedonia and Thrace	8 (4.7)
	Attica	35 (20.7)
	North Aegean	1 (0.6)
	Western Greece	10 (5.9)
	Western Macedonia	5 (3)
	Epirus	1 (0.6)
	Thessaly	15 (8.9)
	Central Macedonia	55 (32.5)
	Crete	10 (5.9)
	South Aegean	2 (1.2)
	Peloponnese	21 (12.4)
	Central Greece	6 (3.6)
Paternal educational level		
	Primary school	8 (4.5)
	Lower high school	25 (14.2)
	Upper high school (Lyceum)	67 (38.1)
	Technical university	24 (13.6)
	University	24 (13.6)
	MSc	19 (10.8)
	PhD	9 (5.1)
Maternal educational level		
	Primary school	8 (4.5)
	Lower high school	9 (5.1)
	Upper high school (Lyceum)	51 (29.0)
	Technical university	46 (26.1)
	University	40 (22.7)
	MSc	15 (8.5)
	PhD	7 (4.0)
		Mean (SD)
Age (years)		17.1 (0.8)
Diploma grade		18.1 (1.4)

**Table 2:** Description of ASQ-14 items.

	Not at All Stressful	A Little Stressful	Moderately Stressful	Quite Stressful	Very Stressful	Mean (SD)	Skewness	Kurtosis
	*n* (%)	*n* (%)	*n* (%)	*n* (%)	*n* (%)			
1. Having arguments at home	49 (27.8)	35 (19.9)	37 (21)	19 (10.8)	36 (20.5)	2.76 (1.48)	0.28	−1.29
2. Feeling a lack of understanding by your parents	19 (10.8)	25 (14.2)	26 (14.8)	40 (22.7)	66 (37.5)	3.62 (1.39)	−0.60	−0.97
3. Being under pressure of study	20 (11.4)	16 (9.1)	44 (25)	48 (27.3)	48 (27.3)	3.5 (1.29)	−0.55	−0.69
4. Having difficulty with some subjects	6 (3.4)	21 (11.9)	62 (35.2)	38 (21.6)	49 (27.8)	3.59 (1.12)	−0.24	−0.76
5. Being subject to compulsory school attendance	36 (20.5)	36 (20.5)	44 (25)	30 (17)	30 (17)	2.9 (1.37)	0.11	−1.17
6. Not having enough time for your boy/girlfriend	86 (48.9)	27 (15.3)	24 (13.6)	22 (12.5)	17 (9.7)	2.19 (1.4)	0.79	−0.79
7. Being dissatisfied with how you look	33 (18.8)	35 (19.9)	42 (23.9)	21 (11.9)	45 (25.6)	3.06 (1.45)	0.04	−1.32
8. Having disagreements between you and your peers	15 (8.5)	31 (17.6)	48 (27.3)	34 (19.3)	48 (27.3)	3.39 (1.29)	−0.23	−1.04
9. Not being listened to by teachers	70 (39.8)	36 (20.5)	28 (15.9)	31 (17.6)	11 (6.3)	2.3 (1.32)	0.56	−1.01
10. Having disagreements between you and your teachers	79 (44.9)	34 (19.3)	37 (21)	17 (9.7)	9 (5.1)	2.11 (1.23)	0.79	−0.46
11. Having a concern about your future	16 (9.1)	11 (6.3)	22 (12.5)	39 (22.2)	88 (50)	3.98 (1.3)	−1.13	0.09
12. Having a lack of freedom	36 (20.5)	50 (28.4)	28 (15.9)	18 (10.2)	44 (25)	2.91 (1.49)	0.24	−1.38
13. Not having enough money to buy the things you want	48 (27.3)	39 (22.2)	23 (13.1)	27 (15.3)	39 (22.2)	2.83 (1.53)	0.20	−1.46
14. Having to take on new family responsibilities as you get older	27 (15.3)	40 (22.7)	42 (23.9)	39 (22.2)	28 (15.9)	3.01 (1.31)	0.00	−1.11

**Table 3:** Exploratory factor analysis results (item loadings, eigenvalues, percentages of variance explained).

	Factor	
	External Pressures and Constraints	Internal Stress and Uncertainty
1. Having arguments at home	0.53	
2. Feeling a lack of understanding by your parents		0.49
3. Being under pressure of study 4. Having difficulty with some subjects		0.84
5. Being subject to compulsory school attendance	0.53	
6. Not having enough time for your boy/girlfriend	0.49	
7. Being dissatisfied with how you look	0.43	
8. Having disagreements between you and your peers	0.40	
9. Not being listened to by teachers	0.57	
10. Having disagreements between you and your teachers	0.48	
11. Having a concern about your future		0.63
12. Having a lack of freedom	0.46	
13. Not having enough money to buy the things you want	0.59	
14. Having to take on new family responsibilities as you get older		0.51
Eigenvalue	2.59	2.04
% variance explained	18.5	14.5

**Table 4:** Cronbach’s alpha coefficients and item-total correlation coefficients.

		Corrected Item-Total Correlation	Cronbach’s Alpha if Item Deleted	Cronbach’s Alpha
External Pressures and Constraints	1. Having arguments at home	0.48	0.73	0.76
	5. Being subject to compulsory school attendance	0.47	0.74	
	6. Not having enough time for your boy/girlfriend	0.45	0.74	
	7. Being dissatisfied with how you look	0.39	0.75	
	8. Having disagreements between you and your peers	0.41	0.74	
	9. Not being listened to by teachers	0.47	0.74	
	10. Having disagreements between you and your teachers	0.35	0.75	
	12. Having a lack of freedom	0.41	0.75	
	13. Not having enough money to buy the things you want	0.53	0.72	
Internal Stress and Uncertainty	2. Feeling a lack of understanding by your parents	0.41	0.70	0.71
	4. Having difficulty with some subjects	0.65	0.56	
	11. Having a concern about your future	0.51	0.63	
	14. Having to take on new family responsibilities as you get older	0.43	0.68	

**Table 5:** Descriptive measures and intercorrelations of ASQ-14 scales.

		Mean (SD)	Pearson’s Correlation Coefficients		
			1.	2.	3.
1.	External Pressures and Constraints	24.44 (7.38)	1.00	0.38 ***	0.92 ***
2.	Internal Stress and Uncertainty	14.19 (3.74)		1.00	0.67 ***
3.	Total ASQ-SF score	42.13 (10.09)			

*** *p* < 0.001.

**Table 6:** ASQ-14 scales by perceived family socioeconomic status.

	Perceived Family Socioeconomic Status				P Student’s *t*-Test
	Low/Middle		High		
	Mean	SD	Mean	SD	
External Pressures and Constraints	23.67	6.56	27.91	9.68	0.003
Internal Stress and Uncertainty	14.47	3.78	12.94	3.32	0.036
Total ASQ-SF score	41.65	9.79	44.28	11.25	0.183

**Table 7:** ASQ-14 scales by gender.

	Gender				P Student’s *t*-Test
	Girls		Boys		
	Mean	SD	Mean	SD	
External Pressures and Constraints	25.21	7.68	22.22	5.96	0.019
Internal Stress and Uncertainty	14.66	3.72	12.82	3.51	0.004
Total ASQ-SF score	43.47	10.07	38.22	9.19	0.002

## Data Availability

At the present, the data presented in this study are available on request from the corresponding author because of ongoing analyses.

## Abbreviations

The following abbreviations are used in this manuscript:

ASQ	Adolescent Stress Questionnaire
EFA	Exploratory Factor Analysis
CFA	Confirmatory Factor Analysis
CFI	Confirmatory Fit Index
TLI	Tucker–Lewis Index
SRMR	Standardized Root Mean Squared Error
RMSEA	Root Mean Square Error of Approximation
PAF	Principal Axis Factoring
KMO	Kaiser-Meyer-Olkin
